# The complete chloroplast genome sequence of *Histiopteris incisa* (Dennstaedtiaceae)

**DOI:** 10.1080/23802359.2018.1491344

**Published:** 2018-07-11

**Authors:** Xinyao Sun, Ziqi Kang, Shanshan Liu, Ruixiang Xu, Zhen Wang, Ting Wang, Yingjuan Su

**Affiliations:** a School of Life Sciences, Sun Yat-sen University, Guangzhou, China;; b College of Life Sciences, Nanjing Agricultural University, Nanjing, China;; c College of Life Sciences, South China Agricultural University, Guangzhou, China;; d Research Institute of Sun Yat-sen University in Shenzhen, Shenzhen, China

**Keywords:** *Histiopteris incisa*, chloroplast genome, phylogenetic analysis

## Abstract

*Histiopteris incisa* is a core member to address the issue of relationship between *Histiopteris* and *Pteris.* Its complete chloroplast genome was generated by *de novo* assembly using Illumina sequencing. The complete chloroplast genome is a quadripartite structure of 16,0567 bp in length, with two inverted repeat regions (IRs) of 27,119 bp each, a large single-copy (LSC) region of 84,897 bp and a small single-copy (SSC) region of 21,432 bp. A total of 132 genes were predicted, involving in 88 protein-coding genes, eight rRNA genes, 35 tRNA genes, and one pseudogene. ML phylogenetic analysis showed that *H. incisa* was closely related to *Pteridium aquilinum subsp. aquilinum*.

*Histiopteris incisa* is only one accepted species of *Histiopteris* in the family Dennstaedtiaceae (Yan et al. 2013). The fern is up to 2 m tall with long-creeping rhizome covered with chestnut-brown scales (Yan et al. 2013). Known as bat's wing fern or water fern, its fronds are soft and green and have deeply lobed segments. The plant is usually found in the moist areas, where it may form extensive colonies. It is widespread across Bhutan, NE India, S Japan, pantropical areas, islands near Antarctica, and Madagascar (Yan et al. 2013). *Histiopteris incisa* is a core member to explore the phylogenetic relationships between *Histiopteris* and *Pteris* due to their morphological similarity (Yan et al. 2013). Hence, acquirement of its whole chloroplast (cp) genome sequence will lay solid foundation to address this issue.

Sample of *H. incisa* was provided by South China Botanical Garden, Chinese Academy of Sciences (23°19’28.21’’N, 113°37’47.46’’E). The specimen is stored in Herbarium of Sun Yat-sen University (SYS; voucher: *SS Liu 20161012*). Total genomic DNA was isolated by Tiangen Plant Genomic DNA Kit (Tiangen Biotech Co., Beijing, China). Average 300 bp Illumina paired-end genomic library was prepared and sequenced on an Illumina Hiseq 2500 platform (Illumina Inc., San Diego, CA). After trimming the sequences using Trimmomatic v0.32 (Bolger et al. 2014), we obtained high quality clean reads and assessed their quality by FastQC v0.10.0 (Andrews 2010). These clean reads were further *de novo* assembled into contigs by Velvet v1.2.07 (Zerbino and Birney 2008). DOGMA (Wyman et al. 2004) and tRNAscan-SE (Schattner et al. 2005) were used to conduct the annotation.

The complete chloroplast genome of *H. incisa* (GenBank accession number: MH319942) is a quadripartite structure of 16,0567 bp in length, with two inverted repeat regions (IRs) of 27,119 bp each, a large single-copy (LSC) region of 84,897 bp and a small single-copy (SSC) region of 21,432 bp. It harbors 132 genes, including 88 protein-coding genes, eight rRNA genes, 35 tRNA genes, and one pseudogene. A total of 18 genes have introns. Among them, *ndhB, rps16, atpF, rpoC1, petB, petD, ndhA, rpl16,* and *rpl2* have a single intron, whereas *ycf3*, *clpP*, and *rps12* contain two introns. Fourteen genes occur as two copies, including four protein- coding genes (*rps12*, *rps7*, *psbA*, and *ycf2*), six tRNA genes (*trnN-GUU*, *trnH-GUG*, *trnI-GAU*, *trnA-UGC*, *trnT-UGU*, and *trnR-ACG*), and four rRNA genes (*rrn4.5*, *rrn5*, *rrn16*, and *rrn23*). The total GC content was 43.0% and the corresponding values in LSC, IRs, and SSC are 41.2%, 46.5%, and 39.1%, respectively.

Phylogenetic analysis was carried out using the complete genome sequence of 14 ferns and *Osmundastrum cinnamomeum* as outgroup by Maximum likelihood (ML) method. After sequence alignment by MAFFT (Katoh et al. 2002), phylogenetic tree was constructed using RAxML (Stamatakis 2014) with 1000 bootstrap replicates. The results revealed that *H. incisa* was closely related to *Pteridium aquilinum subsp. aquilinum* (Figure 1). The complete chloroplast genome of *H. incisa* provides powerful data to further elucidate phylogenetic relationships of dennstaedtioid ferns.

**Figure 1.**
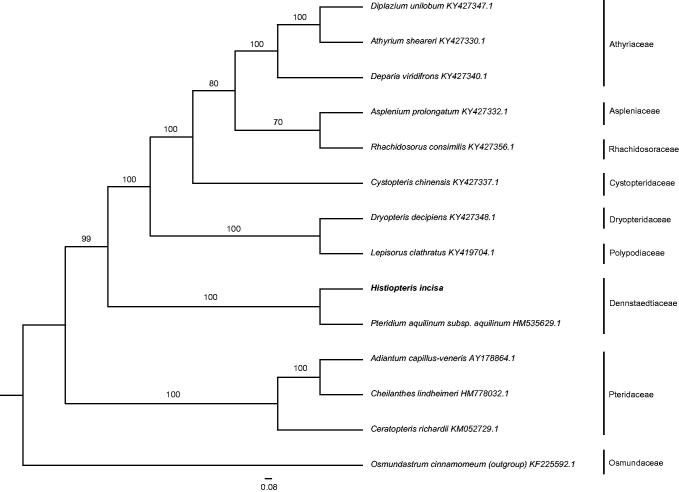
ML phylogenetic tree of *Histiopteris incisa* with 14 ferns *and Osmundastrum cinnamomeum* as outgroup based on complete chloroplast genome sequences. Numbers in the nodes are support values with 1000 bootstrap replicates.
